# Flexible responses to stage‐specific offspring threats

**DOI:** 10.1002/ece3.5832

**Published:** 2019-12-21

**Authors:** Sarah Britton, Barbara Ballentine

**Affiliations:** ^1^ Western Carolina University Cullowhee North Carolina; ^2^ University of Arizona Tucson Arizona

**Keywords:** clutch size, life history, nest competition, parental care, provisioning rates, reproductive strategy

## Abstract

When caring for their young, parents must compensate for threats to offspring survival in a manner that maximizes their lifetime reproductive success. In birds, parents respond to offspring threats by altering reproductive strategies throughout the breeding attempt. Because altered reproductive strategies are costly, when threats to offspring are limited, parents should exhibit a limited response. However, it is unclear if response to offspring threat is the result of an integrated set of correlated changes throughout the breeding attempt or if responses are a flexible set of dissociable changes that are stage‐specific. We test these hypotheses in a system where house wrens (*Troglodytes aedon*) compete for nesting cavities with Carolina chickadees (*Poecile carolinensis*) by usurping and destroying their nests during the early stage of the breeding attempt (the egg stage). Due to the specificity of the house wren threat, we can test whether parental responses to an offspring threat show flexibility and stage specificity or if parental strategies are an integrated and persistent response. We monitored nests in a natural population to compare life history traits of chickadees nesting in boxes that were in the presence of house wrens to chickadees nesting in boxes that did not overlap with house wrens. Carolina chickadees that nested near house wrens laid significantly smaller clutch sizes (early change in reproductive strategy) but did not alter nestling provisioning or nestling stage length (late change in reproductive strategy), suggesting that chickadees respond in a flexible and stage‐specific manner to the threat of house wrens. By responding only when a threat is highest, parents minimize the cost of antithreat responses. Our study suggests that parents can respond in subtle and nuanced ways to offspring threats in the environment and specifically alter reproductive behaviors at the appropriate stage.

## Graphical abstract

In this study, we ask how breeding parents respond to a stage‐specific threat to their offspring. We find that the presence of a threat is associated with changes in parental reproductive strategy only during the stage when the threat is relevant. We find support for the hypothesis that parents have a flexible and stage‐specific response to offspring threat.

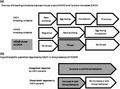

## 1 INTRODUCTION

Parental investment is a complex suite of behaviors that are subject to competing demands on parents as they attempt to care for current offspring, conserve resources for future reproduction, and ensure their own health while dealing with environmental stresses. The need to compensate for environmental stresses, such as the reduction in resources to feed offspring or increasing threat to offspring from parasites, competitors and predators, is an important selective force on the evolution of parental behavior. In many animals, merely detecting predators in the environment can cause parents to adjust reproductive strategies (Lima, 1998; Martin & Briskie, 2009). Reduction in parental care can help conceal offspring (Lima, 2009; Lissåker & Kvarnemo, 2006) or allow parents to reduce the cost of raising young that have low probability of survival (Ibáñez‐Álamo et al., 2015; Lima, 1987). Any strategy that involves reduction in time spent caring for offspring or reduction in investment toward offspring may have significant costs for both parents and offspring in the current breeding attempt (Lissåker & Kvarnemo, 2006; Scheuerlein & Gwinner, 2006). Because threats to offspring are not always consistent across time, we ask hereif parents can adjust reproductive strategies only during the times when a threat is present to reduce the impact of these costs.

Threats to offspring from predation are a primary cause of nest failure in birds and thus an important selective force on the evolution of parental behavior (Martin, 1995). Threats from predation can result in costly alterations in parental behavior throughout nesting (i.e., both egg and nestling stages) because general offspring predators are often relevant throughout the entire breeding attempt (e.g., Fontaine & Martin, 2006; Zanette, White, Allen, & Clinchy, 2011). In the presence of a threat, parents can alter behavior that results in reduced clutch size (Doligez & Clobert, 2003; Eggers, Griesser, Nystrand, & Ekman, 2005; Hua, Sieving, Fletcher, & Wright, 2014; Zanette et al., 2011), reduced clutch mass (Fontaine & Martin, 2006), changes in incubation behavior (Conway & Martin, 2000; Ferretti, Llambías, & Martin, 2005; Ibáñez‐Álamo & Soler, 2012; Massaro, Starling‐Windhof, Briskie, & Martin, 2008), and reduced nestling provisioning (Chalfoun & Martin, 2010; Dudeck, Clinchy, Allen, & Zanette, 2018; Fontaine & Martin, 2006; Pretelli, Isacch, & Cardoni, 2016; Sofaer, Sillett, Peluc, Morrison, & Ghalambor, 2012; Yoon, Kim, Joo, & Park, 2016; Zanette et al., 2011). Increased time off the nest during incubation results in eggs with decreased embryo mass, reduced residual yolk, and reduced growth efficiency (Olson, Vleck, & Vleck, 2006) and decreased nestling provisioning is associated with slower nestling growth and poorer quality nestlings (Scheuerlein & Gwinner, 2006; Thomson et al., 2006; Zanette et al., 2011). Altering an integrated suite of antipredator behaviors across the nesting attempt might be favored because offspring exposure to predators is so costly.

Other types of heterospecific interactions, such as parasitism and interference competition, may also lead to significant threats to offspring survival when adult heterospecifics remove or destroy eggs or nestlings. For example, brood parasites can kill eggs and nestlings, impacting all stages of nesting (Soler, Pérez‐Contreras, & Soler, 2017), and nest site competitors can influence reproductive success of many species (Deng & Zhang, 2016; Finch, 1990; Goldshtein, Markman, Leshem, Puchinsky, & Charter, 2018). Behavioral strategies to protect offspring from predators should be effective against parasitism and interference competition (e.g., Ghalambor & Martin, 2000). However, because antipredator behavior can be so costly, it may be beneficial for parents to respond flexibly to threats that are limited in time or scope to maximize lifetime reproductive success. For example, some interference competitors may pose a threat to eggs but not to nestlings (Belles‐Isles & Picman, 1986). While some studies have found that parents adjust the amount of investment in offspring between the egg and nestling stage (Heaney & Monaghan, 1995; Monaghan, Nager, & Houston, 1998), others have suggested that proximate mechanisms link the amount of investment between these two stages (Bründl et al., 2019). Thus, it remains unclear if changes in parental strategies to avoid offspring threats are an integrated set of traits or if they are dissociable and flexible. Behavioral flexibility that allows parents to alter strategies only when the threat is high to offspring could mitigate the trade‐offs associated with threat reduction behaviors. In this study, we test whether a threat that occurs only early during the breeding attempt results in threat reduction behaviors throughout the nesting cycle or if threat reduction behaviors are restricted to the stage when the threat is highest.

**Figure 1:**
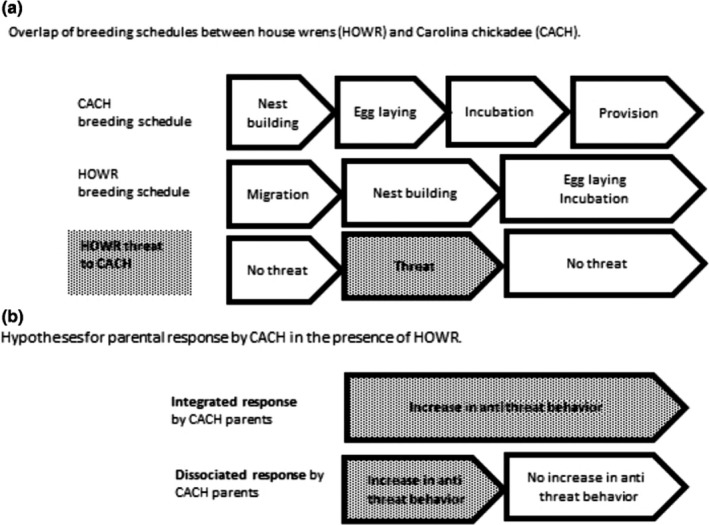
(a) Graphical representation of the relative timing of breeding schedules in Carolina chickadee (CACH) and house wren (HOWR) in western North Carolina. HOWR presents a threat to CACH only early in the nesting attempt. (b) Hypotheses tested in this study: If antithreat reproductive response in CACH is dissociable and parents can adjust strategies per threat level, then we should observe changes in reproductive strategy in early nesting only. If antithreat behavior in CACH is integrated, then we should observe changes in reproductive strategy in early and late nest stages

House wrens are aggressive interference competitors that compete for nesting space by destroying nests of other species (Finch, 1990; Kattan, 2016; Pribil & Picman, 1991; Quinn & Holroyd, 1989). During territory establishment, adult house wrens usurp cavities from other nesting birds, and in some systems this occurs primarily during the egg stage of their competitors, representing a stage‐specific threat to offspring (Belles‐Isles & Picman, 1986). Nest competition between house wrens (*Troglodytes aedon*) and Carolina chickadees (*Poecile carolinensis*) is an ideal study system in which to test whether changes in reproductive strategy are an integrated or flexible set of traits because house wrens are a stage‐specific threat to Carolina chickadee offspring (Figure 1a). When house wrens are present, house wren nest destruction is a primary cause of Carolina chickadee nest failure (Doherty & Grubb, 2002, this study), so we expect the presence of wrens to induce a threatreduction response (i.e., change in reproductive strategy) in nesting chickadees. If chickadee strategies show flexibility and stage specificity, we expect to see that parents in the presence of house wrens will alter their reproductive strategy during the egg stage (i.e., clutch size and incubation behavior) and not the nestling stage (i.e., nestling provisioning and nestling stage length; Figure 1b). Specifically, we predict that parents will decrease clutch size (Doligez & Clobert, 2003; Eggers et al., 2005; Hua et al., 2014; Zanette et al., 2011) or clutch mass (Fontaine & Martin, 2006). We also predict parents will increase off‐bout length, presumably to decrease activity around the nest (Chalfoun & Martin, 2010; Conway & Martin, 2000; Ferretti et al., 2005; Massaro et al., 2008) and decrease incubation attentiveness (Ibáñez‐Álamo & Soler, 2012; Zanette et al., 2011). Alternatively, if Carolina chickadees show an integrated response, we expect to see that parents in the presence of house wrens will alter their reproductive strategy in both the egg and the nestling stage, even though house wrens are primarily a threat only during the former (Figure 1b). Specifically, we predict that in addition to egg stage changes, parents will also decrease nestling provisioning (Chalfoun & Martin, 2010; Dudeck et al., 2018; Fontaine & Martin, 2006; Pretelli et al., 2016; Sofaer et al., 2012; Yoon et al., 2016; Zanette et al., 2011) and decrease nestling stage length which may reduce time at risk in the nest (Hua et al., 2014; Remeŝ & Martin, 2002; Yoon et al., 2016). We discriminate between the predictions of a dissociable response versus the predictions of an integrated response by measuring and comparing life history traits of free‐living chickadees naturally nesting in boxes that are near nesting house wrens to chickadees nesting in boxes that are not.

## 2 METHODS

### 2.1 Study species

Carolina chickadees and house wrens, both cavity nesters that readily use nest boxes, overlap in range and habitat preferences, and thus, interactions between them are common (Johnson, 2014; Mostrum, Curry, & Lohr, 2002). Carolina chickadees, residents in the southeast United States, typically lay one clutch (range 3–7 eggs) per season and usually do not renest after failure (Mostrum et al., 2002). Mean life expectancy of chickadees is approximately 2 years (Mostrum et al., 2002). House wrens, migrants in the southeast United States, historically only overlapped in the northern portion of the chickadee range during breeding (Johnson,^2014^; Mostrum et al., 2002). However, the house wren breeding range is currently expanding further south (Sauer et al., 2014), and during this study, house wrens nested in areas where they were previously undetected (Ballance, 2018; S. B., personal observations). House wrens occupy multiple cavities each season because they build “dummy” nests (nests that are never actively used) in addition to the primary nest (Alworth, 1996; Dubois & Getty, 2003). House wrens gain access to multiple cavities by usurping and destroying nests of current occupants (Belles‐Isles & Picman, 1986; Finch, 1990; Kattan, 2016; Pribil & Picman, 1991; Quinn & Holroyd, 1989). Nest destruction consists of poking holes in eggs of current occupants and sometimes removing eggs and nesting material from the cavity.

### 2.2 Study sites

We studied populations of Carolina chickadees in 2017 using nest boxes that were primarily established by the Highlands Plateau Audubon Society in Macon and Jackson Counties, North Carolina. Two hundred and fifteen nest boxes were distributed in parks, schools, and residential neighborhoods, ranging in elevation from 593 to 1,261 m a.s.l. Placement of nest boxes was sporadic because most boxes were installed by Highlands Plateau Audubon Society as part of a nest box program initiated during the winter of 2014–2015, but participation in the project meant that boxes were being continually added each year, and we installed an additional 50 boxes in January 2017. Both Carolina chickadees and house wrens nested across the range of elevations and in all habitat types (open fields, wooded areas, and human impacted areas). Across all elevations and habitat types, we observed both overlap of chickadees and wrens as well as chickadees nesting in the absence of wrens. Thus, we are reasonably confident that placement and occupancy of nest boxes across a broad patchwork of habitat was random with respect to major habitat features.

Boxes had external dimensions of 5.5 cm length × 5.5 cm width × 29 cm height. Due to a previous study, nest boxes had holes that were either 3.5 cm or 4.5 cm in diameter. Cavity entrance size may influence parenting strategies such as incubation rhythm (Morosinotto, Thomson, & Korpimäki, 2013) or provisioning rates (Yoon et al., 2016), so we accounted for cavity entrance size during statistical analysis.

### 2.3 House wren surveys

We quantified house wren presence by conducting point count surveys from each nest box once a week from March (before expected return of house wrens) through May (after all chickadee nests were initiated) between 8 a.m. and 12 p.m. House wrens were marked as present if seen or heard during at least one survey within a distance that may have reasonably been in a house wren territory (Johnson, 2014). In some cases, house wrens were marked as present if not observed during a point count survey but were seen or heard during nest checks (30.9% of boxes with house wrens present). Because we visited all boxes multiple times per week and because house wrens are conspicuous displayers (in 54.8% of boxes with house wrens present, wrens were seen or heard during multiple surveys), we are confident that we were able to accurately determine presence or absence of house wrens at sites. It was rare to find signs of nesting house wrens without previous auditory or visual detection (4.8% of boxes with house wrens present).

House wrens were first detected in the study area on April 13, and the average date of first detection near a box was April 24. House wren detection overlapped with onset of chickadee egg laying: First egg dates for chickadees in the study area ranged from April 2 to May 19, and the average first egg date was April 20.

### 2.4 Egg stage

We checked boxes once per week starting late February 2017 until chickadee nesting material was observed, then observations increased to two to three times per week to determine the first egg date. Chickadees lay one egg per day in the morning until the clutch is complete (Mostrum et al., 2002); therefore, if we found a nest with more than one egg, we back‐counted to calculate the day that the first egg was laid. We measured clutch mass as soon as possible upon clutch completion (1–2 after the last egg was laid) using a digital scale and calculated average egg mass per clutch as a measurement of investment early in the reproductive attempt.

We installed two Thermochron iButtons (Maxim Integrated) in the nest box prior to clutch completion to determine onset of incubation and incubation rhythm (following Cooper & Mills, 2005; Hartman & Oring, 2006). iButtons are an effective way to monitor nests full time without disturbing the nests since iButtons do not affect nest survival, hatching success, or abandonment (Hartman & Oring, 2006). We placed iButtons just below the nest cup to measure approximate incubation temperatures and in the upper corner of the nest box to measure ambient temperatures in the box. We set the iButtons to record temperature every 5 min, which allowed 6 days worth of data collection and minimized disturbance to the nest to download data and reset iButtons. We checked nests daily starting at the estimated hatch date and removed iButtons upon hatching. For nests where we missed the hatch date, we estimated the date by assuming incubation started the day the last egg was laid and lasted 12 days, the reported average for the species (Mostrum et al., 2002).

We analyzed iButton data with Raven Pro 1.4 (Cornell Lab of Ornithology) and Rhythm (Cooper & Mills, 2005), a program that selects incubation off‐bouts based on a set of parameters. In a few cases, females moved iButtons during incubation such that iButtons were buried in the cup as opposed to being closer to the surface. We used more sensitive parameters to detect off‐bouts when we observed that iButtons were buried upon retrieval (Table 1). As suggested by other authors (Cooper & Mills, 2005), we visually inspected the off‐bout periods that were selected by Rhythm. Spurious off‐bouts, such as off‐bouts selected during the night, we removed before calculating averages. We calculated the average off‐bout length for each nest by including all iButton data from the incubation period, but excluding the off‐bout directly following iButton installment or replacement because it likely reflects a response to human disturbance. We calculated attentiveness as the percentage of on‐bout minutes in total daytime minutes (from first off‐bout to last off‐bout). Validation of iButton data with video recordings of incubation confirm that off‐bouts detected by iButtons measure actual off‐bouts that occur during incubation (Ballance, 2018).

**Table 1:** High‐sensitivity and low‐sensitivity iButton parameters used in Rhythm for off‐bout selection. High‐sensitivity parameters were used when the iButton was buried deep in the nest cup; while low‐sensitivity parameters were used, when the iButton was exposed in the nest cup

Parameter	High sensitivity	Low sensitivity
Minimum off‐bout duration	5 min	5 min
Minimum off‐bout depth (Change in temperature)	1°	2°
Minimum initial slope (cooling)	0.05°/min	0.1°/min
Time‐out (cooling)	30 min	30 min
Minimum initial slope (warming)	0.01°/min	0.05°/min
Time‐out (warming)	30 min	30 min
Maximum final slope (warming)	0.005°/min	0.005°/min

### 2.5 Nestling stage

We installed LawMate cameras (3.5 cm length × 2 cm width × 0.5 cm height; Annandale, VA) on the ceilings inside nest boxes to capture nestling provisioning footage on day seven (*n* = 5), eight (*n* = 28), or nine (*n* = 17) posthatching. We collected 4 hr of video footage between 8:30 a.m. and 2:30 p.m. (*n* = 50 nests, which includes all possible nests with sufficient lighting or without technical failure). We scored the videos for the number of parental trips to the nest. All parental trips to the nest were counted as provisioning trips because previous research indicates that parental trips are an adequate estimate of food delivery in other passerines (Gilby, Mainwaring, Rollins, & Griffith, 2011; McCarty, 2002). We calculated both total parental trip rate (number of trips per hour) and a per nestling rate (number of trips per hour per nestling).

Nestlings were weighed and measured on day 12 of the nestling stage, a few days before expected fledging, to reduce risk of interfering with the fledging process. On this day, we also reinstalled iButtons using the same methods as mentioned above to help determine fledge date. Active nests were visited frequently around predicted fledge dates, starting approximately 16 days after hatching, (Mostrum et al., 2002), but for nests where we missed fledging, we used iButton data to determine fledge date (when there was a drop in nest temperature outside of normal temperature fluctuations). iButtons accurately reflect the day which the last nestling fledges (B. Ballentine, unpublished data). The total length of the nestling stage was calculated using hatch date and fledge date.

### 2.6 Reproductive success

Reproductive success was calculated both as a binary value (whether or not at least one nestling fledged) and in terms of number of nestlings fledged. We assumed that the number of nestlings present on the day 12 visit was the number of nestlings that successfully fledged unless shown otherwise (e.g., dead nestling found). We assumed nests were destroyed by house wrens if eggs were found uneaten on the ground or in the nest with holes, whereas nest failure was recorded as predation if eggs or nestlings were completely absent.

### 2.7 Statistical analysis

We performed statistical analyses in R version 3.3.2 (R Core Team, 2016). The presence of house wrens was scored as a binary variable. All predictions were analyzed with general linear models using the lm() function from base R, unless otherwise noted. It was confirmed that all data adhered to model assumptions and were normally distributed by running diagnostic plots in R. Clutch size and egg size models had house wren presence as the predictor variable and first egg date (as a Julian date value) and elevation as covariates, because both factors may have an effect (Badyaev, 1997; Badyaev & Ghalambor, 2001; Verhulst, Balen, & Tinbergen, 1995). For the egg size model, clutch size was also included as a covariate. Mean off‐bout length and nest attentiveness models had house wren presence as the predictor variable and first egg date and elevation as covariates, as well as hole size of the nest box (Morosinotto et al., 2013), which varied among our boxes. First egg date and elevation were included as covariates as proxies for temperature which can influence incubation behavior (Ardia, Pérez, & Clotfelter, 2010).

Both total and per nestling feeding rate models had house wren presence as the predictor variable with brood size and nest box hole size (Yoon et al., 2016) as covariates. Nestling age had no effect on total (*F*_1,47_ = 1.47, *p* = .23) or per nestling (*F*_1,47_ = 0.73, *p* = .40) provisioning rates, and video start time had no effect on total (*F*_1,47_ = 1.49, *p* = .17) or per nestling (*F*_1,47_ = 1.11, *p* = .30) provisioning rates, so these variables were not included in final provisioning models. Nestling stage length was analyzed with house wren presence as the predictor variable and elevation and first egg date as covariates (Badyaev, 1997).

All three nestling body measurements (mass, wing length, and tail length) were analyzed with linear mixed models using function lme() from package nlme (Pinheiro, Bates, DebRoy, Sarkar, & Team, 2012) with house wren presence as the predictor variable and nest as a random factor.

Reproductive success was first analyzed as a binary measure in relation to house wren presence with a chi‐square test of independence. Then, using only successful nests, we tested whether number of fledglings was influenced by house wren presence.

### 2.8 Ethical approval

This study was conducted in accordance with the ethical standards for animal welfare of the Institutional Animal Care Committee at Western Carolina University IACUC No. RAUP 2016‐001.

## 3 RESULTS

We monitored 215 nest boxes with a total of 103 nesting attempts in 97 different boxes by Carolina chickadees throughout the 2017 breeding season. Fifty‐three nests were successful, and 50 attempts were failures. Six of the unsuccessful nesting attempts may have resulted in renesting in the same box, although this could not be confirmed since birds were not banded (i.e., it is possible the second nesting attempt in the box may have been a different pair). Seventeen nest failures were due to house wren destruction (16 during the egg stage and one during the early nestling stage), 16 failures were due to abandonments (12 during the egg stage, and four during the nestling stage), 14 failures were due to predation (five during the egg stage and nine during the nestling stage), and three failures were due to other causes including bear destruction of boxes (two) and human destruction of a box (one).

### 3.1 Egg stage

**Figure 2:**
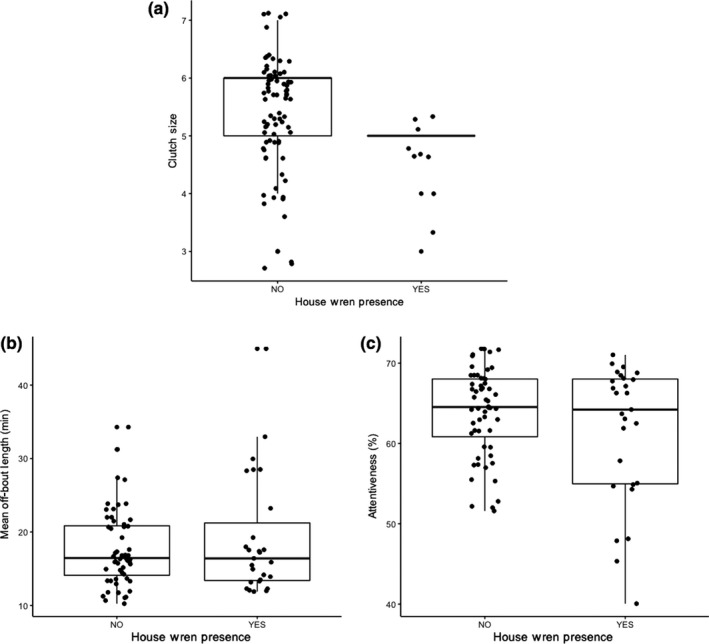
Chickadees showed changes in clutch size but not incubation behavior in response to house wren presence. House wren presence was associated with (a) smaller clutch sizes, but not (b) mean off‐bout length, or (c) incubation attentiveness

In the presence of house wrens, chickadees laid smaller clutches (present: $\bar{x}$ = 5.00 eggs ± 0.15 *SE*, *n* = 28 vs. absent: $\bar{x}$ = 5.47 eggs ± 0.12, *n* = 59, *F*_1,82_ = 6.37, *p* = .01, Figure 2a), although egg mass did not differ (present: $\bar{x}$ = 1.02 g ± 0.02, *n* = 28 vs. absent: $\bar{x}$ = 1.02 g ± 0.01, *n* = 58, *F*_1,80_ = 2.37, *p* = .13).

House wren presence was not associated with changes in mean off‐bout length (present: $\bar{x}$ = 19.06 min ± 1.56, *n* = 27 vs. absent: $\bar{x}$ = 17.64 min ± 0.69, *n* = 56, *F*_1,77_ = 1.35, *p* = .25, Figure 2b) or incubation attentiveness (present: $\bar{x}$ = 61.50% ± 1.65, *n* = 27 vs. absent: $\bar{x}$ = 63.88% ± 0.73, *n* = 56, *F*_1,77_ = 1.59, *p* = .21, Figure 2c). Chickadees in the presence of house wrens showed more variation in both mean off‐bout length (*SD* = 8.12 vs. 5.14) and attentiveness (*SD* = 8.57 vs. 5.46). The difference in variance of attentiveness was significant (Levene's test: *F*_1,81_ = 4.87, *p* = .03), although difference in variance of off‐bout length was not (Levene's test: *F*_1,81_ = 2.38, *p* = .13).

### 3.2 Nestling stage

**Figure 3:**
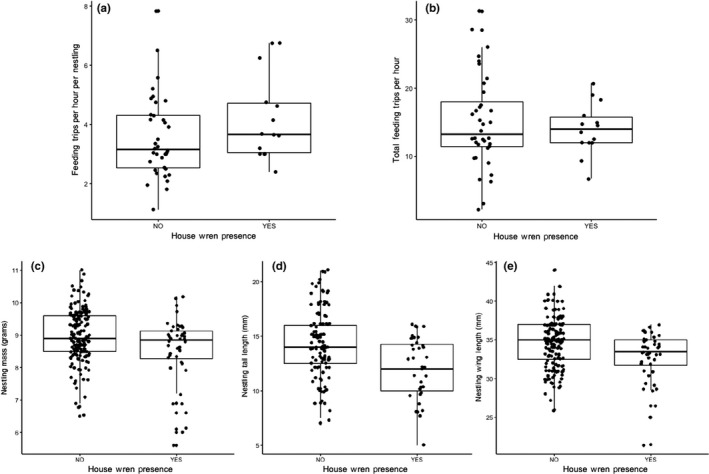
Chickadees did not alter reproductive strategy later in the nesting attempt. House wren presence was not associated with (a) total feeding trips per hr or (b) per nestling feeding trips per hr. House wren presence was not associated with three measures of nestling size: (c) mass, (d) tail length, and (e) wing length

House wren presence was not associated with changes in per nestling provision rate (present: $\bar{x}$ = 4.20 trips per nestling per hr ± 0.39, *n* = 14 vs. absent: $\bar{x}$ = 3.55 trips per nestling per hr ± 0.23, *n* = 36, *F*_1,47_ = 0.62, *p* = .44, Figure 3a) or total provisioning rate (present: $\bar{x}$ = 14.02 trips/hr ± 1.01, *n* = 14 vs. absent: $\bar{x}$ = 14.94 trips/hr ± 1.14, *n* = 36, *F*_1,47_ = 0.06, *p* = .82, Figure 3b). There was a positive effect of brood size on total provisioning (*F*_1,47_ = 31.23, *p* < .001, *r* ^2^ = 0.40) and a negative effect of brood size on per nestling provisioning (*F*_1,47_ = 6.54, *p* = .01, *r* ^2^ = 0.14).

House wren presence was not associated with differences in nestling mass (present: $\bar{x}$ = 8.53 g ± 0.16, *n* = 44 vs. absent: $\bar{x}$ = 8.99 g ± 0.06, *n* = 164, *χ* ^2^ = 1.39, *df* = 1, *p* = .24, Figure 3c), tail length (present: $\bar{x}$ = 12.09 mm ± 0.49, *n* = 35 vs. absent: $\bar{x}$ = 14.29 mm ± 0.29, *n* = 116, *χ* ^2^ = 2.42, *df* = 1, *p* = .12, Figure 3d), or wing length (present: $\bar{x}$ = 32.74 mm ± 0.49, *n* = 44 vs. absent: $\bar{x}$ = 34.69 mm ± 0.26, *n* = 164, χ^2^ = 3.35, *df* = 1, *p* = .07, Figure 3e). House wren presence was not associated with differences in nestling stage length (present: $\bar{x}$ = 16.89 days ± 0.42, *n* = 9 vs. absent: $\bar{x}$ = 16.96 days ± 0.21, *n* = 35, *F*_1,40_ = 0.19, *p* = .66).

### 3.3 Reproductive success

**Figure 4:**
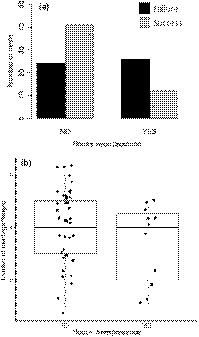
House wren presence was (a) significantly associated with overall chickadee nest failure, but (b) house wren presence was not associated with number of fledglings from successful nests

Reproductive success was significantly dependent on house wren presence (*χ* ^2^ = 8.31, *df* = 1, *p* < .01, Table 2, Figure 4a). However, house wren presence was not associated with number of fledglings from successful nests (present: $\bar{x}$ = 3.36 fledglings ± 0.47, *n* = 11 vs. absent: $\bar{x}$ = 4.05 fledglings ± 0.22, *n* = 39, *F*_1,48_ = 2.02, *p* = .17, Figure 4b).

**Table 2:** Contingency table of values for chi‐square test of independence between house wren presence and overall nest success

	Failed nest	Successful nest
House wren present	26	12
House wren absent	24	41

## 4 DISCUSSION

In breeding birds, presence of a threat is often associated with changes in reproductive strategy throughout the nesting attempt including changes in clutch size, incubation rhythm, nestling provisioning, and nestling development (Chalfoun & Martin, 2010; Ferretti et al., 2005; Fontaine & Martin, 2006). In this study, we investigated whether parental responses to an offspring threat were an integrated set of correlated changes throughout the breeding attempt or flexible and dissociable changes during specific stages by monitoring Carolina chickadee response to house wren competition, a stage‐specific threat. During the 2017 breeding season, house wrens were a major cause of nest failure for chickadees in our study site via nest usurpations (34%), but these almost exclusively occurred during the egg stage (95%). Consistent with the hypothesis of a flexible response to offspring threats, parental changes in reproductive strategy were limited to early in the nesting attempt when house wren threat was highest and most relevant. We show that in the presence of house wrens, chickadees laid smaller clutch sizes, but did not alter strategies during the nestling stage, such as nestling provisioning and nestling stage length.

The reduction of clutch size in a high‐risk nesting attempt is a well‐established pattern and is thought to occur because clutch reduction allows parents to distribute risk among multiple nesting attempts or trade‐off for a future reproductive attempt (Eggers et al., 2005; Ferretti et al., 2005; Hua et al., 2014; Martin, 1995; Olsen, Felch, Greenberg, & Walters, 2008; Zanette et al., 2011). Because Carolina chickadees are single brooded and rarely renest (Mostrum et al., 2002), it is possible that by reducing clutch sizes, chickadees are trading off reproductive investment for increased adult survival to the next breeding season, and thus higher lifetime reproductive success despite reduced seasonal success. In a great tit population that is also single brooded, clutch size was reduced in the presence of an offspring predator, and smaller clutch sizes were associated with increased adult survival (Julliard, McCleery, Clobert, & Perrins, 1997). The mechanisms for the costs of reproduction are becoming better understood; for example, reproductive effort can accelerate senescence via oxidative stress, hormonal regulation, or immune function (Harshman & Zera, 2007; Wiersma, Selman, Speakman, & Verhulst, 2004). A proximate mechanism that may also explain smaller clutch sizes is that direct interactions with house wrens (i.e., nest defense) during oogenesis results in decreased female foraging and thus smaller investment in the clutch. Furthermore, smaller clutch sizes allow for earlier onset of incubation, resulting in a shorter time that the eggs are at risk in the nest (Skutch, 1949).

An alternate explanation is that other environmental differences, such as human disturbance, habitat type, or food abundance may account for both house wren presence and smaller clutch sizes. For example, a previous study found that house wren nest usurpation was primarily associated with edge habitat for chickadees (Doherty & Grubb, 2002). Although we did not explicitly measure habitat parameters at nest boxes, nest boxes were arranged randomly across the environment such that areas of chickadee and wren overlap as well as areas without overlap were found across the range of elevations and habitat types in our study (see Section 2). Future studies should include experimental manipulations that more precisely isolate the effect of house wrens. If house wren presence influences the distribution of chickadees, then it is possible that poorer quality chickadees are relegated to nesting areas that overlap with house wrens, which may be another explanation for smaller clutch sizes in the presence of house wrens. However, because we did not find other measures of decreased parental investment (i.e., incubation and provisioning) in the presence of house wrens, this is not likely the case in our study. Additionally, because most chickadees settle before house wren arrival, it is not as likely that quality plays a role.

Undoubtedly, predators in our study area, which accounted for some nest failures, may have influenced parental investment and behavior. While this may account for some of the variation in our data, we are not aware of any other stage‐specific predators. For example, black rat snakes (*Pantherophis obsoletus*), a common predator that was observed depredating nests on multiple occasions, are a predator of both eggs and nestlings. Additionally, we found that chickadee nest failure due to predation was not any more likely to happen in nests with house wren presence than in nests without house wrens presence (*χ* ^2^ = 0.72, *df* = 1, *p* = .40).

Average incubation off‐bout length and incubation attentiveness were not significantly associated with house wren presence, although interestingly, there was a larger variance in these behaviors in the presence of house wrens. This may indicate that some, but not all, chickadees are responding to the threat of house wrens. There are a few potential explanations for this variation. First of all, changes in reproductive strategy may have a time lag, such that responses to a particular threat occur in later broods (Chalfoun & Martin, 2010; Julliard et al., 1997). It is possible that some chickadees have not had experience with house wrens due to their recent range expansion (Sauer et al., 2014), and that newly exposed individuals may alter reproductive strategies in a future season. Future studies should address parental experience; a long‐term study may reveal whether or not age or parental experience with house wren threat influences reproductive strategy, especially incubation behavior. Alternatively, due to the short life span of chickadees, it may be more common for first year birds to respond more strongly to house wren threat to conserve resources for the following season, while second year birds do not decrease investment since it may be their terminal reproductive attempt.

It is also possible that variation in incubation behavior is driven by proximity to house wrens, such that chickadees closer to house wren territories, or close to multiple territories, alter behavior differently or more dramatically than those further away. Due to small sample sizes and timing constraints, we were not able to explicitly account for varying levels of house wren threat.

Our data show that chickadees did not alter reproductive strategy later in the breeding attempt, consistent with the idea that response to house wren threat is flexible and stage‐specific. While other studies show that presence of a general offspring predator (i.e., a predator that is also a threat to nestlings) may influence feeding rate (Chalfoun & Martin, 2010; Fontaine & Martin, 2006; Pretelli et al., 2016; Sofaer et al., 2012; Yoon et al., 2016; Zanette et al., 2011), we found no such differences. We found instead that variation in number of both total feeding trips and per nestling feeding trips was mostly explained by brood size: Larger broods had more total trips but fewer trips per nestling.

Other studies have found that presence of a threat, such as a predator that is a danger throughout nesting, induces changes in the length of the nestling stage; presumably, an accelerated nestling stage decreases the time that undeveloped nestlings are at risk in the nest (Hua et al., 2014; Yoon et al., 2016). We found no evidence that the nestling stage was accelerated in the presence of house wrens, despite existence of variation in the length of the nestling stage. A shorter nestling stage may result in nestlings that are smaller upon fledging, putting them at greater risk of predation outside the nest (Scheuerlein & Gwinner, 2006; Thomson et al., 2006; Zanette et al., 2011). Thus, by not responding to the threat of house wrens by accelerating the nestling stage, chickadees are avoiding an unnecessary cost.

Although nestling size did not differ significantly among chickadees, our data do show a trend toward smaller nestlings in the presence of house wrens, and marginally nonsignificantly so for wing length. There are factors besides provisioning rates that influence mass at time of fledging, such as stress during laying (Coslovsky & Richner, 2011). It is possible that the trend of decreased nestling size may be due to the effects of stress on the laying females in the presence of house wrens. Alternatively, food load or quality, which we were not able to explicitly measure, may also help explain these differences.

House wren usurpations decreased chickadee reproductive success, an effect that was likely exacerbated by the fact that chickadees are single brooded and have a low mean life expectancy. For those nests that persisted, we were unable to detect a cumulative effect on success in the presence of house wrens—there was no significant difference in terms of number of offspring fledged. However, on average, chickadees in the presence of house wrens fledged fewer young. It is possible that we were unable to detect a significant pattern due to a small sample size, especially for successful nests near house wrens. We had a sample size of 50 successful nests, but a power analysis suggests that we would need a sample size of approximately 136 nests to detect a significant reduction in reproductive success of nests near house wrens. Whether or not the pattern is significant, this reduction in success is not as severe as those reported in other studies due to parental response to presence of a threat (e.g., 40% decrease in number of fledglings under predator threat, Zanette et al., 2011, although more severe treatments could also account for this). Thus, the flexibility to respond to a stage‐specific threat only when necessary may decrease the fitness consequences related to changes in reproductive strategy. This may be especially important for single brooded and short‐lived birds such as Carolina chickadees.

Our study suggests that breeding chickadees can respond in subtle ways to stage‐specific offspring threats from house wrens and alter reproductive behaviors at the appropriate stage. A flexible and stage‐specific response to this offspring threat is notable because it may minimize the negative fitness consequences for both offspring and parents. Understanding the nuance and flexibility of how parents respond to offspring threats sheds light on patterns of parental investment and trade‐offs, and may help explain variation in life history strategies in different contexts.

## CONFLICT OF INTERESTS

The authors declare they have no competing interests.

## AUTHOR CONTRIBUTIONS

Both authors contributed to the design of the project, the field work, and the writing of the manuscript. Data analyses were conducted by Sarah Britton.

## Data Availability

Field data and iButton Data are available on Mendeley. https://doi.org/10.17632/y2f3wv9fv2.2

## References

[ece35832-bib-0001] Alworth, T. (1996). An experimental test of the function of sticks in the nests of house wrens. The Condor, 98, 841–844. 10.2307/1369866

[ece35832-bib-0002] Ardia, D. R. , Pérez, J. H. , & Clotfelter, E. D. (2010). Experimental cooling during incubation leads to reduced innate immunity and body condition in nestling tree swallows. Proceedings of the Royal Society B: Biological Sciences, 277, 1881–1888. 10.1098/rspb.2009.2138 PMC2871872 20147326

[ece35832-bib-0003] Badyaev, A. V. (1997). Avian life history variation along altitudinal gradients: An example with cardueline finches. Oecologia, 111, 365–374. 10.1007/s004420050247 28308131

[ece35832-bib-0004] Badyaev, A. V. , & Ghalambor, C. K. (2001). Evolution of life histories along elevational gradients: Trade‐off between parental care and fecundity. Ecology, 82, 2948–2960. 10.1890/0012-9658(2001)082[2948:EOLHAE]2.0.CO;2

[ece35832-bib-0005] Ballance, T. (2018). Effects of nest quality on incubation and reproductive success in Carolina chickadees (*Poecile**carolinensis*). Western Carolina University.

[ece35832-bib-0006] Belles‐Isles, J.‐C. , & Picman, J. (1986). House wren nest‐destroying behavior. The Condor, 88, 190–193. 10.2307/1368914

[ece35832-bib-0007] Bründl, A. C. , Sorato, E. , Sallé, L. , Thiney, A. C. , Kaulbarsch, S. , Chaine, A. S. , & Russell, A. F. (2019). Experimentally induced increases in fecundity lead to greater nestling care in blue tits. Proceedings of the Royal Society B: Biological Sciences, 286, 20191013. 10.1098/rspb.2019.1013 PMC6599988 31238840

[ece35832-bib-0008] Chalfoun, A. D. , & Martin, T. E. (2010). Parental investment decisions in response to ambient nest‐predation risk versus actual predation on the prior nest. The Condor, 112, 701–710. 10.1525/cond.2010.090242

[ece35832-bib-0009] Conway, C. J. , & Martin, T. E. (2000). Evolution of passerine incubation behavior: Influence of food, temperature, and nest predation. Evolution, 54, 670–685. 10.1111/j.0014-3820.2000.tb00068.x 10937242

[ece35832-bib-0010] Cooper, C. B. , & Mills, H. (2005). New software for quantifying incubation behavior from time‐series recordings. Journal of Field Ornithology, 76, 352–357. 10.1648/0273-8570-76.4.352

[ece35832-bib-0011] Coslovsky, M. , & Richner, H. (2011). Predation risk affects offspring growth via maternal effects. Functional Ecology, 25, 878–888. 10.1111/j.1365-2435.2011.01834.x

[ece35832-bib-0012] Deng, W. , & Zhang, K. (2016). Breeding habitat characteristics and nest survival of yellow‐rumped flycatcher (*Ficedula* *zanthopygia*) in natural tree‐cavities. Russian Journal of Ecology, 47, 194–199. 10.1134/S1067413616020065

[ece35832-bib-0013] Doherty, P. F., Jr. , & Grubb, T. C. Jr (2002). Nest usurpation is an ‘edge effect’ for Carolina chickadees *Poecile* *carolinensis* . Journal of Avian Biology, 33, 77–82. 10.1034/j.1600-048X.2002.330112.x

[ece35832-bib-0014] Doligez, B. , & Clobert, J. (2003). Clutch size reduction as a response to increased nest predation rate in the collared flycatcher. Ecology, 84, 2582–2588. 10.1890/02-3116

[ece35832-bib-0015] Dubois, N. S. , & Getty, T. (2003). Empty nests do not affect female mate choice or maternal investment in house wrens. The Condor, 105, 382–387. 10.1093/condor/105.2.382

[ece35832-bib-0016] Dudeck, B. P. , Clinchy, M. , Allen, M. C. , & Zanette, L. Y. (2018). Fear affects parental care, which predicts juvenile survival and exacerbates the total cost of fear on demography. Ecology, 99, 127–135. 10.1002/ecy.2050 29030965

[ece35832-bib-0017] Eggers, S. , Griesser, M. , Nystrand, M. , & Ekman, J. (2005). Predation risk induces changes in nest‐site selection and clutch size in the Siberian jay. Proceedings of the Royal Society B: Biological Sciences, 273, 701–706. 10.1098/rspb.2005.3373 PMC1560074 16608689

[ece35832-bib-0018] Ferretti, V. , Llambías, P. E. , & Martin, T. E. (2005). Life‐history variation of a neotropical thrush challenges food limitation theory. Proceedings of the Royal Society B: Biological Sciences, 272, 769–773. 10.1098/rspb.2004.3039 PMC1602047 15870039

[ece35832-bib-0019] Finch, D. M. (1990). Effects of predation and competitor interference on nesting success of house wrens and tree swallows. The Condor, 92, 674–687. 10.2307/1368686

[ece35832-bib-0020] Fontaine, J. , & Martin, T. (2006). Parent birds assess nest predation risk and adjust their reproductive strategies. Ecology Letters, 9, 428–434. 10.1111/j.1461-0248.2006.00892.x 16623728

[ece35832-bib-0021] Ghalambor, C. K. , & Martin, T. E. (2000). Parental investment strategies in two species of nuthatch vary with stage‐specific predation risk and reproductive effort. Animal Behaviour, 60, 263–267. 10.1006/anbe.2000.1472 10973729

[ece35832-bib-0022] Gilby, A. J. , Mainwaring, M. C. , Rollins, L. A. , & Griffith, S. C. (2011). Parental care in wild and captive zebra finches: Measuring food delivery to quantify parental effort. Animal Behaviour, 81, 289–295. 10.1016/j.anbehav.2010.10.020

[ece35832-bib-0023] Goldshtein, A. , Markman, S. , Leshem, Y. , Puchinsky, M. , & Charter, M. (2018). Nest‐site interference competition with house sparrows affects breeding success and parental care in great tits. Journal of Ornithology, 159, 667–673. 10.1007/s10336-018-1541-4

[ece35832-bib-0024] Harshman, L. G. , & Zera, A. J. (2007). The cost of reproduction: The devil in the details. Trends in Ecology & Evolution, 22, 80–86. 10.1016/j.tree.2006.10.008 17056152

[ece35832-bib-0025] Hartman, C. A. , & Oring, L. W. (2006). An inexpensive method for remotely monitoring nest activity. Journal of Field Ornithology, 77, 418–424. 10.1111/j.1557-9263.2006.00073.x

[ece35832-bib-0026] Heaney, V. , & Monaghan, P. (1995). A within‐clutch trade‐off between egg production and rearing in birds. Proceedings of the Royal Society of London. Series B: Biological Sciences, 261, 361–365.

[ece35832-bib-0027] Hua, F. , Sieving, K. E. , Fletcher, R. J., Jr. , & Wright, C. A. (2014). Increased perception of predation risk to adults and offspring alters avian reproductive strategy and performance. Behavioral Ecology, 25, 509–519. 10.1093/beheco/aru017

[ece35832-bib-0028] Ibáñez‐Álamo, J. , Magrath, R. D. , Oteyza, J. , Chalfoun, A. , Haff, T. , Schmidt, K. , … Martin, T. (2015). Nest predation research: Recent findings and future perspectives. Journal of Ornithology, 156, 247–262. 10.1007/s10336-015-1207-4

[ece35832-bib-0029] Ibáñez‐Álamo, J. D. , & Soler, M. (2012). Predator‐induced female behavior in the absence of male incubation feeding: An experimental study. Behavioral Ecology and Sociobiology, 66, 1067–1073. 10.1007/s00265-012-1357-9

[ece35832-bib-0030] Johnson, L. S. (2014). House Wren (*Troglodytes aedon*), version 2.0 In Poole A. F. (Ed.), The Birds of North America. Ithaca, NY, USA: Cornell Lab of Ornithology 10.2173/bna.380

[ece35832-bib-0031] Julliard, R. , McCleery, R. H. , Clobert, J. , & Perrins, C. M. (1997). Phenotypic adjustment of clutch size due to nest predation in the great tit. Ecology, 78, 394–404. 10.1890/0012-9658(1997)078[0394:PAOCSD]2.0.CO;2

[ece35832-bib-0032] Kattan, G. H. (2016). Heterospecific infanticidal behavior by southern house wrens (*Troglodytes * *aedon* *musculus*) suggests nest site competition. The Wilson Journal of Ornithology, 128, 899–903.

[ece35832-bib-0033] Lima, S. L. (1987). Clutch size in birds: A predation perspective. Ecology, 68, 1062–1070. 10.2307/1938378

[ece35832-bib-0034] Lima, S. L. (1998). Nonlethal effects in the ecology of predator‐prey interactions. BioScience, 48, 25–34. 10.2307/1313225

[ece35832-bib-0035] Lima, S. L. (2009). Predators and the breeding bird: Behavioral and reproductive flexibility under the risk of predation. Biological Reviews, 84, 485–513. 10.1111/j.1469-185X.2009.00085.x 19659887

[ece35832-bib-0036] Lissåker, M. , & Kvarnemo, C. (2006). Ventilation or nest defense—parental care trade‐offs in a fish with male care. Behavioral Ecology and Sociobiology, 60, 864–873. 10.1007/s00265-006-0230-0

[ece35832-bib-0037] Martin, T. E. (1995). Avian life history evolution in relation to nest sites, nest predation, and food. Ecological Monographs, 65, 101–127. 10.2307/2937160

[ece35832-bib-0038] Martin, T. E. , & Briskie, J. V. (2009). Predation on dependent offspring: A review of the consequences for mean expression and phenotypic plasticity in avian life history traits. Annals of the New York Academy of Sciences, 1168, 201–217. 10.1111/j.1749-6632.2009.04577.x 19566709

[ece35832-bib-0039] Massaro, M. , Starling‐Windhof, A. , Briskie, J. V. , & Martin, T. E. (2008). Introduced mammalian predators induce behavioural changes in parental care in an endemic New Zealand bird. PLoS ONE, 3, e2331 10.1371/journal.pone.0002331 18523640 PMC2396284

[ece35832-bib-0040] McCarty, J. P. (2002). The number of visits to the nest by parents is an accurate measure of food delivered to nestlings in tree swallows. Journal of Field Ornithology, 73, 9–15. 10.1648/0273-8570-73.1.9

[ece35832-bib-0041] Monaghan, P. , Nager, R. , & Houston, D. (1998). The price of eggs: Increased investment in egg production reduces the offspring rearing capacity of parents. Proceedings of the Royal Society of London. Series B: Biological Sciences, 265, 1731–1735. 10.1098/rspb.1998.0495

[ece35832-bib-0042] Morosinotto, C. , Thomson, R. L. , & Korpimäki, E. (2013). Plasticity in incubation behaviour under experimentally prolonged vulnerability to nest predation. Behaviour, 150, 1767–1786. 10.1163/1568539X-00003119

[ece35832-bib-0043] Mostrum, A. M. , Curry, R. L. , & Lohr, B. (2002). Carolina chickadee (*Poecile**carolinensis*), version 2.0 In Poole A. F., & Gill F. B. (Eds.), The birds of North America. Ithaca, NY: Cornell Lab of Ornithology 10.2173/bna.636

[ece35832-bib-0044] Olsen, B. J. , Felch, J. M. , Greenberg, R. , & Walters, J. R. (2008). Causes of reduced clutch size in a tidal marsh endemic. Oecologia, 158, 421 10.1007/s00442-008-1148-1 18825417

[ece35832-bib-0045] Olson, C. R. , Vleck, C. M. , & Vleck, D. (2006). Periodic cooling of bird eggs reduces embryonic growth efficiency. Physiological and Biochemical Zoology, 79, 927–936. 10.1086/506003 16927239

[ece35832-bib-0046] Pinheiro, J. , Bates, D. , DebRoy, S. , Sarkar, D. , & Team, R. C. (2012). nlme: Linear and nonlinear mixed effects models. R package version 3.

[ece35832-bib-0047] Pretelli, M. G. , Isacch, J. P. , & Cardoni, D. A. (2016). Variation in parental care in the spectacled tyrant *Hymenops* *perspicillatus* is associated with increased nest predation in grassland fragments. Journal of Ornithology, 157, 451–460. 10.1007/s10336-015-1300-8

[ece35832-bib-0048] Pribil, S. , & Picman, J. (1991). Why house wrens destroy clutches of other birds: A support for the nest site competition hypothesis. The Condor, 93, 184–185. 10.2307/1368624

[ece35832-bib-0049] Quinn, M. S. , & Holroyd, G. L. (1989). Nestling and egg destruction by house wrens. The Condor, 91, 206–207. 10.2307/1368165

[ece35832-bib-0050] R Core Team (2016). R: A language and environment for statistical computing [online]. Vienna, Austria: R Foundation for Statistical Computing.

[ece35832-bib-0051] Remeŝ, V. , & Martin, T. E. (2002). Environmental influences on the evolution of growth and developmental rates in passerines. Evolution, 56, 2505–2518. 10.1111/j.0014-3820.2002.tb00175.x 12583590

[ece35832-bib-0052] Sauer, J. R. , Hines, J. E. , Fallon, J. E. , Pardieck, K. L. , Ziolkowski, J. D. J. , & Link, W. A. (2014). The North American breeding bird survey, results and analysis 1966–2013 version 01.30.2015. Laurel, MD: USGS Patuxent Wildlife Research Center.

[ece35832-bib-0053] Scheuerlein, A. , & Gwinner, E. (2006). Reduced nestling growth of East African stonechats *Saxicola* *torquata* *axillaris* in the presence of a predator. Ibis, 148, 468–476. 10.1111/j.1474-919X.2006.00549.x

[ece35832-bib-0054] Skutch, A. F. (1949). Do tropical birds rear as many young as they can nourish? Ibis, 91, 430–455. 10.1111/j.1474-919X.1949.tb02293.x

[ece35832-bib-0055] Sofaer, H. R. , Sillett, T. S. , Peluc, S. I. , Morrison, S. A. , & Ghalambor, C. K. (2012). Differential effects of food availability and nest predation risk on avian reproductive strategies. Behavioral Ecology, 24, 698–707. 10.1093/beheco/ars212

[ece35832-bib-0056] Soler, M. , Pérez‐Contreras, T. , & Soler, J. (2017). Brood parasites as predators: Farming and mafia strategies In Soler M. (Ed.), Avian brood parasitism (pp. 271–286). Cham, Switzerland: Springer.

[ece35832-bib-0057] Thomson, R. L. , Forsman, J. T. , Mönkkönen, M. , Hukkanen, M. , Koivula, K. , Rytkönen, S. , & Orell, M. (2006). Predation risk effects on fitness related measures in a resident bird. Oikos, 113, 325–333. 10.1111/j.2006.0030-1299.14376.x

[ece35832-bib-0058] Verhulst, S. , Van Balen, J. , & Tinbergen, J. (1995). Seasonal decline in reproductive success of the great tit: Variation in time or quality? Ecology, 76, 2392–2403. 10.2307/2265815

[ece35832-bib-0059] Wiersma, P. , Selman, C. , Speakman, J. R. , & Verhulst, S. (2004). Birds sacrifice oxidative protection for reproduction. Proceedings of the Royal Society of London. Series B: Biological Sciences, 271, S360–S363. 10.1098/rsbl.2004.0171 15504018 PMC1810045

[ece35832-bib-0060] Yoon, J. , Kim, B.‐S. , Joo, E.‐J. , & Park, S.‐R. (2016). Nest predation risk influences a cavity‐nesting passerine during the post‐hatching care period. Scientific Reports, 6, 31989 10.1038/srep31989 27553176 PMC4995485

[ece35832-bib-0061] Zanette, L. Y. , White, A. F. , Allen, M. C. , & Clinchy, M. (2011). Perceived predation risk reduces the number of offspring songbirds produce per year. Science, 334, 1398–1401. 10.1126/science.1210908 22158817

